# Elderly depression in front-line health services:A descriptive and evaluative cross-sectional study

**DOI:** 10.1192/j.eurpsy.2023.1978

**Published:** 2023-07-19

**Authors:** K. Mahfoudh, A. Ouertani, A. Aissa, S. Maddouri, U. Ouali, R. Jomli

**Affiliations:** “A” Department, Razi Hospital, Manouba, Tunisia

## Abstract

**Introduction:**

Depression is a common pathology in the elderly, often unrecognized and mostly affiliated to the consequences of aging,especially in front-line services. It induces somatic and functional impact and even a suicidal risk.

When unrecognized or neglected, depression can reduce life expectancy and increases the use of healthcare and the institutionalization.

**Objectives:**

-Determine the point-prevalence of depression in the elderly in a front-line heath service in Tunis.

- Identify risk factors of depression in the elderly.

**Methods:**

A descriptive and evaluative cross-sectional study on a sample of patients aged 65 or more, in a tunisian front-line service of general health care regardless of the medical reason.

Sociodemographic and clinical forms along with the PHQ-9 scale (Patient health questionnaire-9)

- validated in tunisian dialect- were used.

**Results:**

Thirty patients have participated in our study (21 men and 9 women). The average age was 73.23 years. Chronic pathologies were found in 96.66% of cases.

The found risk factors are: female sex in 70% of cases, loneliness and isolation in 10% of cases, widowhood in 50% of cases, grieving in 6.6% of cases and somatic comorbidity in 96 .66% of cases.

The overall prevalence of depressive symptoms was 53.33%. This is correlated with advanced age and female sex (57.14% in women vs 33.33% in men).

The depressive symptomatology found, was mild in 18.5% of cases, average in 62.5% of cases and moderately severe in 18.5% of cases.

**Conclusions:**

Depression is a frequent pathology in the elderly with multiple risk factors. The aging of the Tunisian population on one hand and the change of the social model (family nucleus) on the other, encourage the early detection of depression in the elderly and the training of the health professionals in order to limit its prevalence.

**Disclosure of Interest:**

None Declared

